# Efficacy and safety of 5% lidocaine patches for postoperative pain management in patients undergoing unilateral inguinal hernia repair: study protocol for a prospective, double-blind, randomized, controlled clinical trial

**DOI:** 10.1186/s13063-022-06700-3

**Published:** 2022-09-11

**Authors:** Hong-min Ahn, Heung-Kwon Oh, Duck-Woo Kim, Sung-Bum Kang, Bon-Wook Koo, Pyung-Bok Lee

**Affiliations:** 1 Department of Surgery, Seoul National University Bundang Hospital, 166 Gumi-ro, Bundang-gu, Seongnam, 13620 South Korea; 2 Department of Anesthesiology and Pain Medicine, Seoul National University Bundang Hospital, 166 Gumi-ro, Bundang-gu, Seongnam, 13620 South Korea

**Keywords:** Topical analgesics, Lidocaine patch, Inguinal hernia repair

## Abstract

**Background:**

Acute postoperative pain is a common complication of inguinal hernia repair. Pain management using local application of anesthetic agents over the skin surrounding the surgical incision may reduce the requirement for other pain medications. Targeted topical analgesics such as 5% lidocaine patches have been known to improve acute and chronic pain. However, the clinical effect of lidocaine patches on postoperative pain after inguinal hernia repair has not been studied, especially in patients undergoing surgery at day surgery units.

**Methods/design:**

This is a single-center, prospective, double-blind, randomized, controlled clinical trial. Participants with unilateral inguinal hernia will be randomized to the lidocaine patch group or the placebo patch group. Based on the randomized allocation sequence, either lidocaine patches or placebo patches will be attached near each participant’s surgical wound after open hernia repair under general anesthesia. Participants will be asked to follow up at our outpatient clinic on the first postoperative day and at 1 week after surgery. The primary outcome is pain intensity, which will be measured using the visual analog scale (VAS) at the time of discharge from the day surgery unit. The secondary outcomes are VAS score at 24 h and 1 week after surgery. We will collect and analyze the participants’ clinical data (amount of intraoperative opioid use, time to recovery, and pain intensity at 30 min after surgery) and demographic characteristics (age, sex, body weight, and height).

**Discussion:**

This trial may not only provide evidence on the efficacy of a 5% lidocaine patch for acute postoperative pain management after unilateral inguinal hernia repair, but also demonstrate the efficacy and safety of the patch for post-discharge pain management.

**Trial registration:**

ClinicalTrials.gov NCT04754451. Registered on February 10, 2021.

**Supplementary Information:**

The online version contains supplementary material available at 10.1186/s13063-022-06700-3.

## Background

Inguinal hernia repair is a commonly performed in general surgery. Acute postoperative pain, which can be moderate to severe in intensity, commonly occurs after open inguinal hernia repair [1, 2]. The intensity of pain after open inguinal hernia repair has been reported to be the highest on the first postoperative day [1]. Untreated postoperative pain or a high pain score within 24 h of surgery may lead to persistent pain after inguinal hernia repair [3, 4]. Patients with prolonged pain have an increased duration of hospitalization and may fail to return to their daily lives [5]. For these reasons, various modalities, such as pain medication and nerve block, have been used for postoperative pain management [3, 6].

Acute postoperative pain is primarily managed with intravenous or oral administration of narcotic or non-narcotic analgesics. However, most of these drugs are systemically administered, which may result in complications such as nausea, vomiting, inhibition of bowel motility, and abdominal discomfort. These adverse effects can occur even after discharge from the hospital, especially in patients undergoing day surgery for inguinal hernia repair.

On the other hand, pain management with topical applied analgesics near the surgical incision site may reduce the incidence of complications and drug interactions [7]. A lidocaine patch is a pain management modality that involves local infiltration of an analgesic. The use of a 5% lidocaine patch has only been approved by the Food and Drug Administration for treating neurologic pain due to herpes zoster infection; it has not been approved for acute postoperative pain management [8].

Several studies have demonstrated the positive effects of lidocaine patches on acute postoperative pain [9, 10]. However, to the best of our knowledge, there are no studies on the clinical effects of lidocaine patches for acute postoperative pain management after inguinal hernia repair, especially in patients admitted to day surgery units. Furthermore, the use of a lidocaine patch may minimize the systemic effects of the analgesic agent and reduce the financial burden on the patient by decreasing the requirement for the extra pain medication. Therefore, we designed this double-blind, randomized, controlled clinical trial to determine the efficacy and safety of a 5% lidocaine patch for postoperative pain management in patients undergoing unilateral inguinal hernia repair at a day surgery unit.

## Methods/design

### Study design

**Fig. 1:**
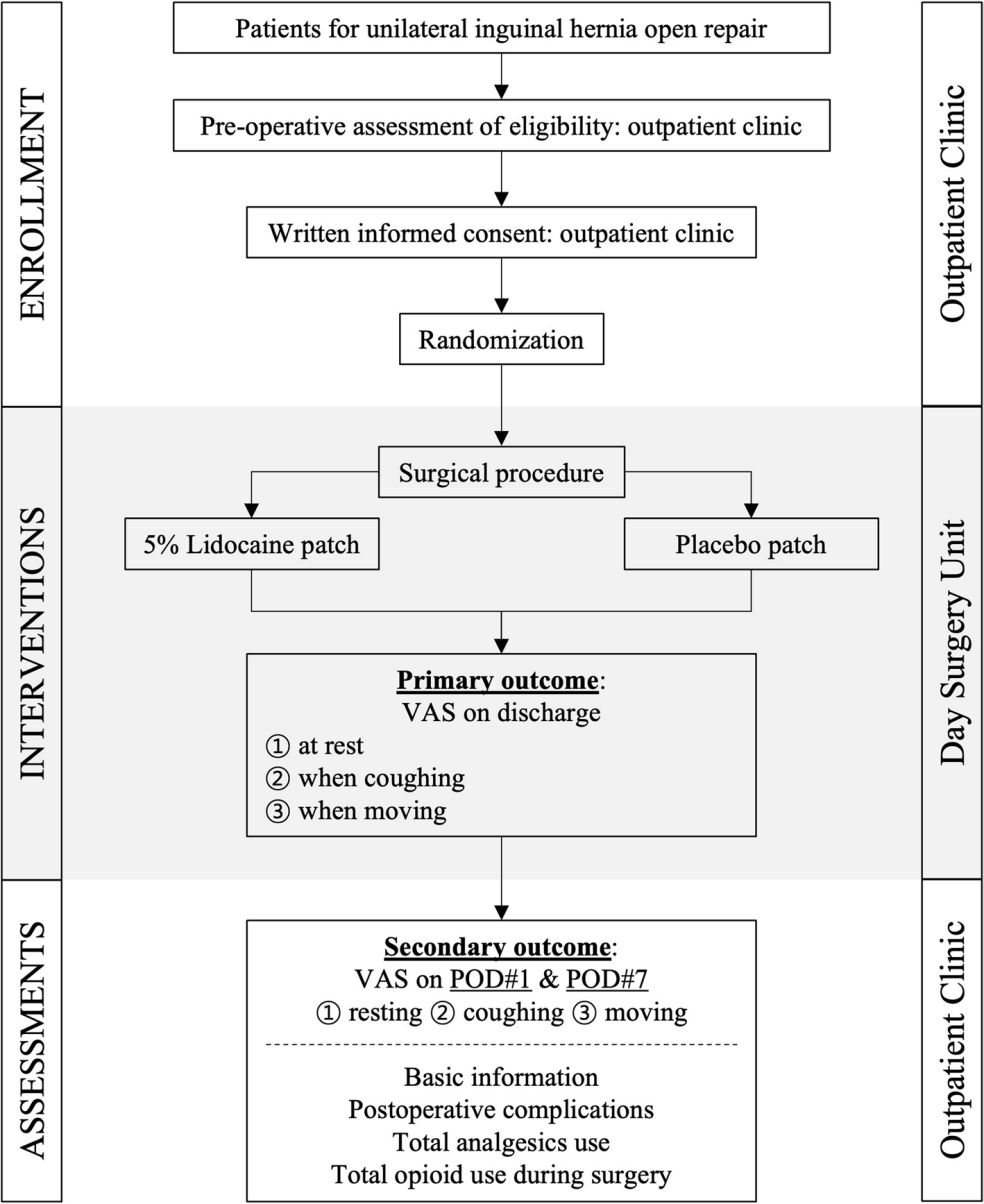
Trial design flowchart. VAS, visual analog scale; POD, postoperative day

This is a single-center, prospective, superiority, double-blind, randomized, controlled clinical trial. This study has been approved by the Institutional Review Board (B-2007-625-006) of Seoul National University Bundang Hospital. Written informed consent to participate will be obtained by the principal investigator at the time of screening for eligibility on the patient’s initial visit to outpatient clinic. After surgery, the experimental group will receive 5% lidocaine patches (Lidotop Cataplasma; SK chemical Co. Ltd, Seongnam, Korea), and the control group will receive placebo patches that were specially made for this study. The comparator is a well-made placebo patch to evaluate not only the efficacy but also the safety of the 5% lidocaine patch. The overview of the study design is shown in Fig. 1.

### Hypothesis

Evaluating the efficacy of a 5% lidocaine patch on acute postoperative pain in a patient undergoing unilateral inguinal hernia repair at a day surgery unit is the main purpose of this study. This prospective, superiority, double-blind, randomized, controlled clinical trial was designed based on the hypothesis that patients who applied the lidocaine patch will have a lower VAS score at discharge after surgery than patients who applied the placebo patch after unilateral inguinal hernia repair at a day surgery unit.

### Inclusion and exclusion criteria

The inclusion criteria are (a) age between 19 and 80 years, (b) American Society of Anesthesiologists risk classification score of I (healthy) or II (mild systemic disease), (c) elective open surgery for unilateral inguinal hernia, and (d) admission to and discharge from the day surgery unit on the day of the surgery itself.

The exclusion criteria are (a) obesity (body mass index > 30 kg/m^2^), (b) renal insufficiency (serum creatinine level ≥ 1.4 mg/dL), (c) liver insufficiency (serum aspartate transferase level ≥ 120 IU/L or serum alanine transaminase level ≥ 120 IU/L), (d) known hypersensitivity to amide-based local anesthetics, (e) use of class I antiarrhythmic medication such as tocainide and mexiletine, and (f) refusal to participate.

The principal investigator of this study will not exclude patients who are likely to participate in this study based on race or socioeconomic status. Every effort will be made to ensure that as many patients as possible who meet the inclusion criteria will be included. Patients will be informed of the purpose of the study so that they can represent the entire population of patients undergoing general anesthesia.

### Sample size calculation

The sample size for the primary outcome (VAS score at the time of discharge) was determined based on a previous study on patients with ventral hernia that compared the VAS scores of a control group (4.8 ± 1.4) with those of a lidocaine patch group (3.1 ± 1.6) and observed a difference of 1.7 [9]. For a one-sided analysis with a type I error (α) of 5% and a power (1-β) of 0.8, the number of participants required per group is 14. Anticipating a dropout rate of 10%, the number of participants required per group is 16, resulting in a total sample size of 32.

### Randomization

We will randomize the participants in a 1:1 ratio to the lidocaine patch group or the placebo patch group. We will achieve block randomization using Random Allocation Software (Isfahan University of Medical Sciences, Isfahan, Iran). A research nurse from the anesthesiology department who is not involved in the study will generate the randomized allocation sequence and deliver the allocated patches. The patches will be given directly to the anesthesiologist and surgeon responsible for applying it at the end of the surgery. The participants and researchers (the anesthesiologist and surgeon) who evaluate the patients’ clinical parameters will be blinded to the group allocation.

### Procedure

**Fig. 2:**
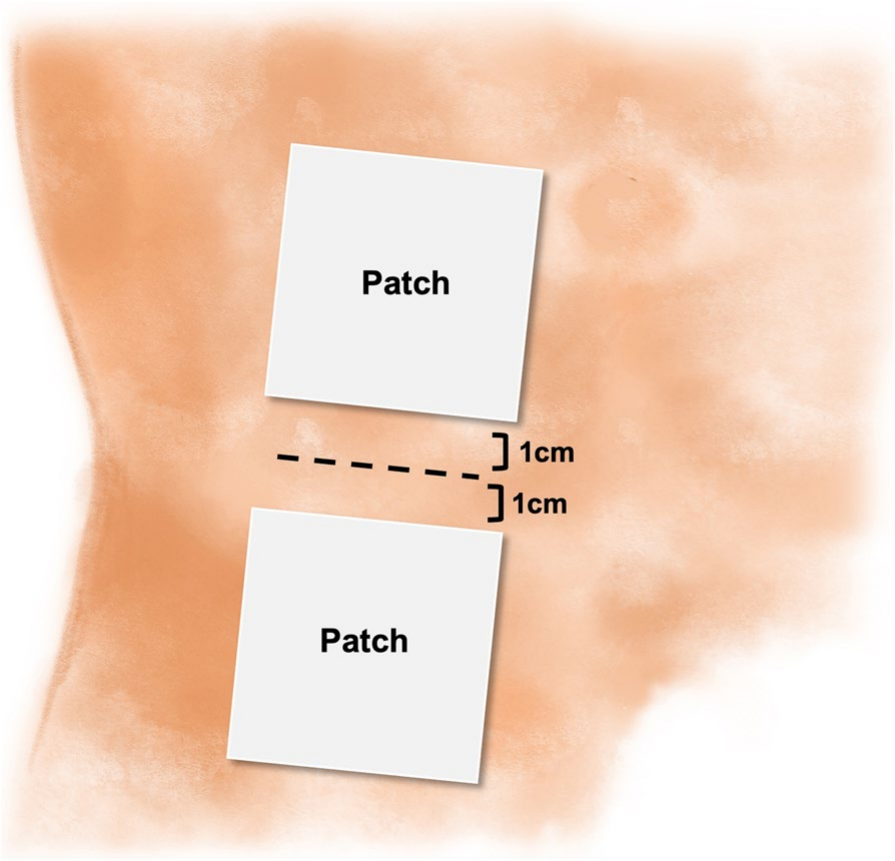
Location of the patch. Irrespective of the type of patch used (5% lidocaine or placebo), one patch will be applied 1 cm above and another 1 cm below the surgical wound

All participants will undergo unilateral inguinal hernia repair under general anesthesia. As pre-treatment, each patient will be injected with 0.03 mg/kg of midazolam on arrival at the operating room. An anesthesiologist will induce and maintain general anesthesia with inhaled anesthetics and opiates using conventional methods. A general surgeon will make a transverse incision of approximately 3 to 4 cm along the skin fold line and perform routine Lichtenstein tension-free mesh inguinal hernia repair. The skin will be closed with subcutaneous interrupted sutures, and skin bond (7 mm × 60 mm; Leukosan SkinLink, BSN medical, Hamburg, Germany) will be applied. After covering the surgical wound with aseptic dressing, lidocaine or placebo patches will be applied near the surgical wound based on the randomized allocation sequence. Each patient will receive two patches, which will be placed 1 cm above and 1 cm below the surgical wound (Fig. 2).

Before discharge from the day surgery unit, we will allow participants to use additional pain medications such as opioids or non-steroidal anti-inflammatory drugs as needed during their recovery from the general anesthesia. Either fentanyl (50 μg; Hana fentanyl citrate, Hana Pharm. Co., Ltd, Seoul, Korea) or ketorolac (30 mg; Trolac, Whan In Pharm. Co., Ltd, Seoul, Korea) will be prescribed. Fentanyl will be prescribed a maximum of three times when a patient experiences pain with a VAS score of > 5. Ketorolac will be prescribed if a patient experiences any adverse effects following fentanyl administration. Oral medications such as tramadol/acetaminophen (37mg/325 mg; Rapicet, Chong Kun Dang Pharm. Co., Seoul, Korea) will be prescribed to each patient at the time of discharge for pain management. However, additional pain medication or interventions outside of the protocol during the study period, which is from the surgery to 1 week after surgery, are prohibited.

This study protocol includes criteria for discontinuing intervention. If a participant withdraws their consent, decides to undergo extra surgery besides the unilateral inguinal hernia repair after random allocation, or if any adverse effects with local anesthetics are shown, they will discontinue the study.

### Outcomes

The primary outcome is pain intensity measured using the VAS at the time of discharge from the day surgery unit, which is approximately 2 or 3 h after the surgery. We will evaluate each patient’s VAS scores in three situations: at rest, when coughing, and when moving. The secondary outcomes are pain intensity at 24 h and 1 week after surgery. We will evaluate each patient’s VAS score when they visit the clinic for wound dressing on the first postoperative day when the pain-relieving effect of the patch has been worn off. At the follow-up visit 1 week after surgery, we will assess the VAS score, the rates of postoperative complications (such as nausea, vomiting, and desaturation), and the total amount of oral combination analgesics used after discharge. To understand the correlation with general anesthesia and lidocaine patch, we will additionally analyze the amount of intraoperative opioid use, time to recovery, and pain intensity at 30 min after surgery as a part of the secondary outcomes. The participants’ demographic characteristics, such as age, sex, body weight, and height, will also be recorded.

### Strategies for study retention

All participants will receive a text message informing them of the date, time, and place for the next outpatient clinic until the end of the study period. In case of no-shows, personal calls to the participant will be made to encourage attendance.

### Data analysis

All statistical analyses will be performed using Statistical Package for the Social Sciences version 25.0 for Windows (IBM, Armonk, NY). The participants’ demographic characteristics will be analyzed using descriptive statistics. After verifying the normality, the Student t-test or Mann–Whitney *U* test will be used to analyze the numerical data. For categorical data, we will use the chi-squared test or Fisher’s exact test. All results with a p-value of less than 0.05 will be considered as significant.

### Data monitoring

The principal investigator will report any unexpected problems or non-compliance with the protocol to the participant in question, the Institutional Review Board of Seoul National University Bundang Hospital, the Ministry of Food and Drug Safety, and the reviewing committee, if necessary. If an adverse drug reaction is the cause of death or is life-threatening, the initial report should be submitted within 7 days. If an adverse drug reaction is not fatal, the initial report should be submitted within 15 days. Minor non-compliance cases should be reported every year on a case-by-case basis.

### Ethics and dissemination

Collected information for research will be stored in a locked file in a laboratory, and access to the research file will be restricted to authorized researchers. Unnecessary personal data will be removed, and information such as patient’s name, resident registration number, and medical chart number should not be recorded. The identifying codes linked to personal information will be managed separately. The records of the participants will be kept confidential and will not be moved to other places, and may be sent, if necessary, to an authorized person for supervision of this study.

We will disseminate the methods and results of our study to the public through social media, presentations at domestic and international congresses on corresponding areas of interest, and by submitting a paper(s) describing our findings to appropriate scientific journals.

## Discussion

Targeted topical analgesics such as 5% lidocaine patches have been known to improve acute and chronic pain [7, 11]. Previous studies have investigated the effects of lidocaine patches on postoperative pain management following various surgeries. Most of these studies included patients with small trocar wounds after laparoscopic surgeries, such as laparoscopic appendectomy, ventral hernia repair, and gynecologic surgery. A prospective study of 40 patients who underwent laparoscopic appendectomy in whom a lidocaine patch was applied at the umbilical trocar site showed that a 5% lidocaine patch might be effective for managing port-site pain [12]. Another study of 30 patients who underwent laparoscopic ventral hernia repair also found that lidocaine patches are effective for postoperative pain management [9]. A study of 40 patients who underwent laparoscopic gynecologic surgery found that patients who received a lidocaine patch had lower VAS scores and additional analgesic requirement than those who did not receive a lidocaine patch [10].

There are a few studies in which a lidocaine patch was used postoperatively for incisions that were longer than a trocar incision. In a randomized controlled trial of 70 patients who underwent radical retropubic prostatectomy with a lower midline incision, a lidocaine patch was applied for 24 h after the surgery, resulting in reduced pain scores and opioid consumption [13]. A similar trial conducted on 28 patients who underwent open gynecological surgery with a midline incision found that postoperative pain reduced with lidocaine patch use [14].

Through these studies, which included randomized clinical trials and meta-analyses, the safety and efficacy of using a 5% lidocaine patch for acute postoperative pain management has been established. However, most of these studies were on patients who received hospital care and patient-controlled analgesia following surgery, in which the patients’ acute pain was rapidly treated by medical staff. However, the lidocaine patch has advantages comparing with other operative pain managements, such as abdominal muscle fascia and peritoneal nerve block and patient-controlled analgesia. It is non-invasive and simple to apply since the lidocaine patch may produce an analgesic effect without a complete sensory blockage by penetrating skin depth of only 8 to 10 mm [15]. The lidocaine patch may also have a great safety profile for application in an outpatient clinical setting for acute postoperative pain.

We have designed this clinical trial to investigate the efficacy of a 5% lidocaine patch for postoperative pain management without additional medical attention. Therefore, we will enroll patients undergoing open unilateral inguinal hernia repair at a day surgery unit. We believe that in addition to demonstrating the efficacy of a 5% lidocaine patch on acute postoperative pain after unilateral inguinal hernia repair, we will be able to provide evidence on its efficacy and safety for post-discharge pain management. In summary, we hypothesize that a 5% lidocaine patch is an effective option for acute postoperative pain management following unilateral inguinal hernia repair, and it can be safely used in an outpatient clinical setting.

## Trial status

This protocol version 1.0 was first posted on February 15, 2021, in Clinicaltrials.gov, without any amendments after then. Patient recruitment commenced in March 2021, and the predicted date of completion of this study is October 2021.

## Supplementary Information

**Additional file 1.**

## Data Availability

Not applicable

## Abbreviations

VAS: Visual analog scale
