# Supplementary material for: Efficacy and safety of 5% lidocaine patches for postoperative pain management in patients undergoing unilateral inguinal hernia repair: study protocol for a prospective, double-blind, randomized, controlled clinical trial
Source: Trials. 2022 Sep 11;23:767. doi: 10.1186/s13063-022-06700-3 (PMC9465907; doi:10.1186/s13063-022-06700-3)
Supplement: Supplementary file 1 — Additional file 1. [file 13063_2022_6700_MOESM1_ESM.pdf]

분당서울대학교병원 Approval
IRB No : B-2007-625-006
심의일 : 2021.08.23
생명윤리심의위원회

별첨 1.

<연구대상자 설명문>

대상자 스크리닝 번호:_______________

연구의 제목 : 단측 서혜부 탈장 봉합 수술을 받는 환자에서 5% 리도카인 패치가 수술 후 통증에 미치는 효과

귀하께 본 연구에 참여하여 주실 것을 요청 드립니다.

본 연구에 참가하실 것을 결정하기 전에 본 연구가 왜 실시되며 무엇을 어떻게 하게 될지 정확하게 이해하는 것이 중요합니다. 아래의 내용은 이번 연구의 내용과 이 연구에 참여하실 경우, 귀하가 하실 역할과 연구의 진행과정 등에 대하여 설명을 드리고자 마련한 것입니다. 충분한 시간을 가지고 본 대상자 설명서를 읽으시고, 원하신다면 가족이나 다른 사람과 상의하셔도 됩니다. 또한, 궁금하신 사항이 있으시면 시험책임자나 다른 시험담당자에게 질문하신 후 심사숙고 하시어 이번 연구 참여여부를 결정하시기 바랍니다. 본 임상시험은 치료목적이 아니며, 단측 서혜부 탈장 봉합 수술을 받는 환자에서 5% 리도카인 패치가 수술 후 통증에 미치는 효과를 평가하기 위한 연구목적으로 수행됩니다.

1. 임상시험의 목적

이 연구의 목적은 단측 서혜부 탈장 봉합 수술을 받는 환자에서 5% 리도카인 패치가 수술 후 통증에 미치는 효과를 평가하기 위한 것입니다. 리도카인 패치는 현재 대상포진 후 신경통 환자에서만 식약처 허가가 되어있고 이번 연구는 수술 후 통증에서도 효과가 있는 지를 보기 위한 검증되지 않는 임상시험입니다.

2. 임상시험용 의약품에 관한 정보 및 시험군 또는 대조군에 무작위배정될 확률

- 시험군: 리도카인 패치를 절개부위 위아래로 1매씩 총 2매 부착합니다.
  대조군: 위약 패치를 절개부위 위아래로 1매씩 총 2매 부착합니다.
- 본 연구는 무작위 배정된 이중 맹검 시험입니다. 이중맹검이란 귀하나 귀하의 담당의사 누구도 귀하가 무작위로 배정될 2가지 투약군 중 어떠한 것을 받게 될지 모르는 것을 의미합니다. 귀하가 어느 군에 배정되는지는 컴퓨터에 의하여 만들어진 난수표에 따라 무작위로 결정되며, 무작위로 배정되는 비율은 1:1로 동일합니다).

분당서울대학교병원 Approval
IRB No : B-2007-625-006
심의일 : 2021.08.23
생명윤리심의위원회

3. 대상자의 임상시험 예상 참여 기간 및 임상시험에 참여하는 대략의 대상자 수

예상 참여기간: 수술 종료부터 수술 후 첫 외래 내원 시(수술 후 1주일)까지.

참여자 수: 총 32명의 대상자가 이 임상시험에 참여하게 될 것입니다.

4. 침습적 시술(侵襲的 施術, invasive procedure)을 포함하여 임상시험에서 대상자가 받게 될 각종 검사나 절차

- 수술종료 시 리도카인 혹은 위약 패치를 절개부위 위아래로 1매씩 총 2매를 12시간동안 부착합니다.
- 수술 후 검사 : 면담을 통한 회복실 퇴실 시(대략 수술 후 30분), 당일수술센터 퇴원 시(대략 수술 후 2시간), 수술 후 24시간(전화통화), 수술 후 1주일 통증평가척도(0-10점의 숫자통증점수)를 측정합니다. 모든 통증척도평가는 휴식시, 기침 시, 움직일 시의 3종류로 측정합니다.
- 퇴원 후 통증 조절: 퇴원 후에는 분당서울대학교 병원에서 서혜부 탈장 수술 후 일반적으로 처방되는 트라마돌을 지참하게 됩니다. 하루에 6시간 간격으로 3번까지 복용이 가능합니다. 부작용으로는 무력증, 피로, 발열, 흉통, 경직, 실신, 금단증상, 현기, 두통, 진전, 운동실조, 경련, 혼미, 복통, 변비, 설사, 소화불량, 방귀, 구내건조, 오심, 구토 등의 증사이 나타날 수 있습니다.

5. 대상자가 준수하여야 할 사항

- 귀하의 건강에 변화가 있거나, 임상시험에 대해 어떠한 우려가 있다면 즉시 연구자에게 알려야 합니다.

6. 임상시험으로 검증하고자 하는 사실

현재 우리나라에서 리도카인 패치는 대상포진 후 신경통에만 적응증으로 되어 있어 수술 후 통증조절에는 허가가 되어있지 않습니다. 리도카인 패치의 적응증을 확대하고 수술 후 통증관리의 새로운 방법으로 효과적일 것이라는 가설을 검증하고자 합니다.

7. 대상자에게 미칠 것으로 예상되는 위험이나 불편

① 패치를 3매 초과하여 부착하는 경우 리도카인의 과량투여로 인한 증상(현기증, 불안, 걱정, 행복감, 착란, 졸음, 이명, 시야흐림, 복시, 구토, 온냉감, 무감각, 연축, 경련, 진전, 의식 불명, 호흡억제, 호흡정지) 이 생길 수 있습니다.

② 국소이상반응: 패치 적용부위에 수포, 멍, 작열감, 탈색소, 피부염, 변색, 부종, 홍

분당서울대학교병원 Approval IRB No : B-2007-625-006 심의일 : 2021.08.23 생명윤리심의위원회

반, 비늘, 자극, 구진, 점출혈, 가려움, 소포가 생기거나 이상 감각이 나타날 수 있습니다. 하지만 이 반응들은 일반적으로 경미하거나 일시적이며, 수분~수시간 내에 자연적으로 소실됩니다.

③ 알레르기반응: 드물기는 하지만, 이 약에 의한 알레르기 반응과 아나필락시스유사반응이 일어날 수 있습니다. 증상은 혈관부종, 기관지연축, 피부염, 호흡곤란, 과민증, 후두경련, 가려움, 쇼크, 두드러기이며, 통상적 방법으로 치료될 수 있습니다.

④ 기타이상반응: 시판 후 조사에서 보고된 인과관계가 밝혀지지 않은 기타 이상반응은 다음과 같습니다: 신경쇠약증, 착란, 방향감장애, 어지럼증, 두통, 감각과민, 감각저하, 현기증, 금속성 맛, 오심, 불안, 통증, 마비, 졸음, 입맛변성, 구토, 시각장애(시야흐림), 홍조, 이명, 진전.

8. 기대되는 이익

리도카인 마취제이며 신경 차단 시 사용되는 약물로 직접약물을 복용하거나 혈관내 주사방법에 비해서 국소적으로 마취제를 수술 절개 부위와 가깝게 직접 침윤시키면 여러 진통제의 약물 상호 작용을 최소화하고 부작용을 감소시킬 수 있으며 비교적 간편하게 부착할 수 있기 때문에 퇴원 후에도 적절한 통증 감소 관리가 될 것으로 예상하고 있습니다. 하지만 이 임상 시험에 참여하는 것이 귀하에게 직접적인 이익이 없을 수도 있습니다. 그러나, 귀하의 참여가 환자의 수술 후 통증 감소 개선에 대해 좀 더 과학적인 정보를 얻게 해 줄 수 있습니다.

9. 대상자가 선택할 수 있는 다른 통증조절 방법 종류 및 그 통증조절 방법의 잠재적 위험과 이익

귀하는 본 임상시험에 참여하지 않을 경우 기존의 통증조절 방법(병원에서는 진통제의 혈관내 주사, 퇴원하여 귀가 후는 진통제의 복용)을 받으실 수 있습니다.

10. 임상시험과 관련한 손상이 발생하였을 경우 대상자에게 주어질 보상이나 치료방법

임상시험 기간 중 의료진은 귀하의 안전을 지키려고 최선을 다해 노력할 것이고 중대한 이상반응 발생시는 빠르고 적절한 조치를 취하여 가능한 한 그 이상반응을 최소화 할 것입니다. 리토카인의 과량 투여(현기증, 불안, 걱정, 행복감, 착란, 졸음, 이명, 시야흐림, 복시, 구토, 온냉감, 무감각, 연축, 경련, 진전, 의식 불명, 호흡억제, 호흡정지), 국소이상반응(수포, 멍, 작열감, 탈색소, 피부염, 변색, 부종, 홍반, 비늘, 자극, 구진, 점출혈, 가려움, 소포가 생기거나 이상 감각), 알레르기반응(혈관부종, 기관지연축, 피부염, 호흡곤란, 과민증, 후

분당서울대학교병원 Approval
IRB No : B-2007-625-006
심의일 : 2021.08.23
생명윤리심의위원회

두경련, 가려움, 쇼크, 두드러기), 기타이상반응(신경쇠약증, 착란, 방향감장애, 어지럼증, 두통, 감각과민, 감각저하, 현기증, 금속성 맛, 오심, 불안, 통증, 마비, 졸음, 입맛변성, 구토, 시각장애(시야흐림), 홍조, 이명, 진전) 등이 일어날 수 있으나 근본적인 치료는 약물이 대사되어 배설이 되어야 하므로 각 증상에 맞춰 약물의 점적 또는 지속 투여를 통하여 증상치료를 할 것입니다.

이 임상시험에 참여하면서 임상시험용 의약품 투여와 관련된 피해가 발생할 경우에는 해당 연구자들이 피해자 보상 규약에 따라 합법적 절차를 거쳐 배상 혹은 보상하게 될 것이며, 이상반응 및 질환 악화의 경우에는 가능한 한 최선의 치료방법으로 치료할 것입니다.

11. 임상시험에 참여함으로써 받게 될 금전적 보상

없음

12. 임상시험에 참여함으로써 대상자에게 예상되는 비용

임상시험에 참여함으로써 추가적으로 발생하는 비용은 없습니다.

13. 자발적 참여 여부

임상시험에 참여하는 것은 전적으로 귀하의 선택에 달려 있습니다.

만일 임상시험에 대한 설명을 듣고 참여에 동의하지 않더라도 전혀 문제가 되지 않습니다. 또한 시험참여에 동의하신 이후라도 귀하가 원하실 경우, 언제라도 임상시험참여 동의를 철회할 수 있으며, 어떠한 불이익이나 손해도 보지 않을 것입니다.

14. 의무기록열람

임상시험 진행 중 및 종료 후에도 임상시험의 모니터요원, 점검을 실시하는 사람, IRB 및 식품의약품안전처장 등이 관계 법령에 따라 연구의 절차와 자료의 품질을 검증하기 위하여 대상자의 신상에 관한 비밀이 보호되는 범위에서 대상자의 연구기록을 열람할 수 있으며, 대상자 서명 동의서에 서명하심으로써 귀하 또는 귀하의 대리인께서는 이러한 자료의 직접열람을 허용함을 의미 합니다.

15. 신상 비밀 보호

본 연구와 관련되지 않은 제3자(예 : 다른 연구자)에게 2차적 사용을 목적으로 개인정보(예 : 개인식별정보, 유전정보 또는 건강에 관한 정보 등 개인에 관한 정보)는 알려지지 않을 것이며 이 연구가 진행되면서 얻어진 귀하의 신상에 대한 모든 기록들은 다른 사람에게

분당서울대학교병원
Approval
IRB No : B-2007-625-006
심의일 : 2021.08.23
생명윤리심의위원회

알려지지 않도록 비밀로 보장될 것입니다. 또한 연구 결과가 출판될 경우에도 귀하의 신상 정보는 비밀상태로 유지될 것입니다.

연구대상자의 개인정보보호 방안

- 본 연구에서 연구대상자를 비식별화 번호로 처리된 연구용 데이터로 수집하는 것을 원칙으로 하며 개인 식별을 할 수 없도록 조치합니다. 환자 개인 정보인 원내 병록번호는 연구자에게 전달하기 전, 임의의 번호를 부여하여 처리 후 수집하는 것을 원칙으로 합니다.
- 본 연구의 본래 목적 외로 의무기록 조사 결과를 이용하거나 타인에게 제공하는 것을 철저하게 금합니다.
- 통계분석자가 대상자의 개인식별정보를 확인 혹은 열람할 수 없습니다.
- 본 연구에서 사용하는 연구용 데이터 원본은 변환 완료 후 폐기하여 개인정보노출의 위험을 미연에 방지하고자 합니다.

생명 윤리법 시행규칙 제 15조에 따라 연구관련 기록은 연구가 종료된 시점부터 3년간 보관하도록 하며, 보관이 지난 문서는 개인정보보호법 시행령 제 16조에 따라 파기합니다.

16. 새로운 정보알림

이 연구가 진행되는 동안에 귀하가 연구참여 지속 여부를 생각하게 될 만한 새로운 사실이나 정보를 시험자가 알게 되면, 언제든지 연구자는 귀하 또는 귀하의 대리인에게 이 사실이나 정보를 알려드릴 것입니다.

17. 임상시험과 대상자의 권익에 관하여 추가적인 정보를 얻고자 하거나 임상시험과 관련이 있는 손상이 발생한 경우에 연락해야 하는 사람

언제라도 본인 또는 대상자의 대리인이 전화면담을 할 수 있으며, 연락할 수 있는 사람은 다음과 같습니다.

임상시험에서 발생한 문제, 우려, 질문에 대하여 상의할 담당자의 연락처

시험 책임자: 이평복 부교수 ☎031-787-7499

연구대상자의 권익에 대한 문제, 우려, 질문이 있을 때 상의할 IRB(생명윤리심의위원회) 또는 임상연구윤리센터 연락처

생명윤리심의위원회(031-787-8801 ~ 8806)

분당서울대학교병원
Approval
IRB No : B-2007-625-006
심의일 : 2021.08.23
생명윤리심의위원회

18. 임상시험 도중 대상자의 임상시험 참여가 중지되는 경우 및 그 사유

귀하의 담당의사는 의학적 이유나 다른 이유로 귀하를 중도탈락 시킬 수도 있습니다.

- 연구대상자의 동의 철회 경우
- 다른 수술과 협진하는 경우
- 국소마취제의 이상반응이 나타나는 경우
- 그 외 시험자의 의견에 따라 임상시험을 진행하는 것이 대상자에게 유익성을 제공하는 것이 아니라고 생각되는 모든 임상적 이상반응 발생시 중도 탈락합니다.

분당서울대학교병원
Approval
IRB No : B-2007-625-006
심의일 : 2021.08.23
생명윤리심의위원회

연구 대상자 동의서

==

1. 본인은 설명문을 읽음과 동시에 담당 의사로부터 구두로 자세히 설명을 듣고 의문 사항에 대해서 충분히 의논하였습니다.

2. 본인은 발생 가능한 위험에 관하여 들었으며 나의 질문에 만족할 만한 답변을 얻었습니다.

3. 본인은 언제든지 중도 탈락을 결정할 수 있고, 이러한 결정이 향후 진료에 있어 불이익을 받지 않을 것을 알고 있습니다.

4. 본인은 이 동의서에 서명함으로써 현행 법률과 규정이 허용하는 범위 내에서 필요한 개인의 정보가 수집된다는 것을 알고 있습니다.

5. 본인은 이 동의서 사본을 받을 것을 알고 있습니다.

==

본인은 담당 의사로부터 사전 설명을 들은 후 이에 자발적으로 동의합니다.

날짜(년/월/일)	________	성명(대상자)	________	서명	______
날짜(년/월/일)	__________	참관인 성명	__________	서명	_______
날짜(년/월/일)	__________	성명(책임/담당 연구자)	_____/_____ _	서명	___/___ _

분당서울대학교병원
Approval
IRB No : B-2007-625-006
심의일 : 2021.08.23
생명윤리심의위원회
